# A Multi-Modal Decision-Level Fusion Framework for Hypervelocity Impact Damage Classification in Spacecraft

**DOI:** 10.3390/s26030969

**Published:** 2026-02-02

**Authors:** Kuo Zhang, Chun Yin, Pengju Kuang, Xuegang Huang, Xiao Peng

**Affiliations:** 1 School of Automation Engineering, University of Electronic Science and Technology of China, Chengdu 611731, China; 2 School of Information and Communication Engineering, University of Electronic Science and Technology of China, Chengdu 611731, China; 3 National Key Laboratory of Aerospace Physics in Fluids, Mianyang 621000, China; emei-126@126.com (X.H.);

**Keywords:** hypervelocity impact, damage classification, multimodal fusion, deep residual network, decision-level fusion

## Abstract

During on-orbit service, spacecraft are subjected to hypervelocity impacts (HVIs) from micrometeoroids and space debris, causing diverse damage types that challenge structural health assessment. Unimodal approaches often struggle with similar damage patterns due to mechanical noise and imaging distance variations. To overcome these physical limitations, this study proposes a physics-informed multimodal fusion framework. Innovatively, we integrate a distance-aware infrared enhancement strategy with vibration spectral subtraction to align heterogeneous data qualities while employing a dual-stream ResNet coupled with Dempster–Shafer (D-S) evidence theory to rigorously resolve inter-modal conflicts at the decision level. Experimental results demonstrate that the proposed strategy achieves a mean accuracy of 99.01%, significantly outperforming unimodal baselines (92.96% and 97.11%). Notably, the fusion mechanism corrects specific misclassifications in micro-cracks and perforation, ensuring a precision exceeding 96.9% across all categories with high stability (standard deviation 0.74%). These findings validate the efficacy of multimodal fusion for precise on-orbit damage assessment, offering a robust solution for spacecraft structural health monitoring.

## 1. Introduction

During the in-orbit operation of spacecraft, it is impossible to avoid the hypervelocity impact (HVI) caused by micrometeoroids and space debris (MMOD) [1,2,3]. Such impacts typically act on the surface and internal units of structures at extremely high incident speeds and transient kinetic energy, causing a wide variety of damages (such as craters, cracks, perforations, etc.) with a large scale range. Moreover, the development patterns and maintenance strategies of different damage types vary significantly [4,5,6,7]. Therefore, precise detection of HVI damage is fundamental, and the development of classification technologies capable of accurately distinguishing different types of damage has become a key technical requirement for supporting the long-term stable service of spacecraft [8,9]. In this context, detection methods based on vibration response analysis and infrared thermography offer promising technical paths to address these challenges, due to their advantages of non-contact, remote, and full-field coverage [10,11,12,13]. Katam et al. [10] proposed a damage detection method based on STFT time–frequency feature extraction and autoencoder dimensionality reduction, achieving high-accuracy damage classification under limited data conditions through time–frequency analysis of vibration signals. Zhang et al. [11] introduced a vibration-based damage detection approach utilizing phase-based motion estimation and convolutional neural networks, which enables precise localization of bolt looseness damage with single-sample training via pixel-level vibration signal extraction from videos. Gao et al. [12] proposed a hypervelocity impact (HVI) damage detection method based on infrared data extraction and multi-objective optimization algorithms. By extracting representative infrared features and reconstructing defect regions, the method effectively improved detection accuracy and efficiency. Yin et al. [13] introduced the Dynamic Multi-Objective Feature Extraction Optimization (DM-FEO) method, which enhances the precision of HVI damage detection through temperature point extraction in infrared thermography and multi-directional prediction algorithms, successfully distinguishing the thermal characteristics of different damage types. Vibration detection is sensitive to changes in the structural frequency response function, making it effective for identifying local stiffness degradation and delamination damage, as well as macro-scale defects [14]. Recent research has further demonstrated that coupling finite element models with metaheuristic optimization algorithms can significantly enhance the precision of damage localization in composite structures [15]. Wang et al. [16] comprehensively reviewed the physical principles, excitation modalities, and applications of multimode infrared thermal-wave imaging in non-destructive testing. In particular, the integration of active infrared imaging with deep learning techniques has been identified as a highly accurate solution for defect detection in complex industrial components [17]. However, it is important to note that these methods primarily serve the detection and preliminary identification of “damage or no damage.” When the task objective shifts to “high-precision classification” of damage types, the inherent limitations of these unimodal methods become evident.

Accurate damage classification of spacecraft, such as identifying crack, perforation, and delamination, is crucial for supporting precision on-orbit maintenance. This task faces three major challenges: first, different damage types exhibit high feature similarity under a single modality, making them difficult to distinguish accurately; second, vibration signals are susceptible to noise interference while infrared features are influenced by imaging distance, leading to poor model stability—a problem also prominent in complex industrial settings, as Tian et al. [18] demonstrated that variable working conditions hinder stable feature extraction; third, the scarcity of annotated on-orbit samples coupled with class imbalance limits the training effectiveness of data-driven models, prompting Yin et al. [19] to employ generative adversarial networks to enhance scarce sample representation. Thus, relying on a single modality fails to achieve both comprehensive coverage and discriminative robustness, particularly under sample-limited conditions required for high-accuracy classification.

To overcome the limitations of unimodal methods, recent research has shifted toward multimodal data fusion for detection [20,21]. These studies demonstrate that integrating complementary multimodal information is an effective strategy for improving performance in complex tasks. However, existing fusion methods primarily focus on feature-level or data-level fusion, requiring precise alignment of heterogeneous data, and they do not explicitly address the critical issue of uncertainty in classification tasks. As a result, their effectiveness is limited when handling challenges such as distinguishing highly similar damage types in fine-grained classification. Data-driven automatic feature learning and classification provide a new technological path for HVI damage detection [22,23,24]. Models, particularly convolutional neural networks (CNNs) and residual networks (ResNet), can learn discriminative features across scales and forms in an end-to-end manner, alleviating the degradation of deep models and enhancing training stability [25]. Ye et al. [26] developed a multi-sensor residual fusion network utilizing double-ring residual and global interactive modules to achieve robust diagnosis under noisy and small-sample conditions; Le-Xuan et al. [27] constructed a hybrid 1DCNN-LSTM-ResNet architecture that effectively captures long-term temporal dependencies and enhances damage detection accuracy. Yan et al. [28] proposed a QCNN-based method for bearing fault diagnosis by fusing audio and vibration signals for high-precision detection in noisy environments.

Furthermore, the combination of multimodality and deep learning shows even better prospects. Xu et al. [29] proposed a multimodal neural network fusion method that integrates large-kernel networks with an attention mechanism, achieving highly robust damage identification in noisy environments. Meshram et al. [30] designed an integrated model utilizing multimodal transformers and hybrid deep learning to optimize pothole detection and repair in diverse environments. Cao et al. [31] proposed a multimodal feature fusion model combining ResNet and GRU to distinguish pseudo defects in ultra-thick stainless-steel welds using Phased Array Ultrasonic Testing. Peng et al. [32] developed a multimodal hybrid neural network, significantly improving damage classification accuracy and data efficiency under complex operational conditions. Research by Zaman et al. [33] and Chen et al. [34] also demonstrated the advantages of multimodal fusion in fault diagnosis. However, the performance of these methods is highly dependent on the scale and quality of training data. In HVI damage scenarios, real labeled data is scarce, especially for infrared images, where image quality is highly dependent on the shooting distance. Distance uncertainty leads to feature distribution shifts, which has become a major bottleneck in applying deep learning methods.

**Figure 1:**
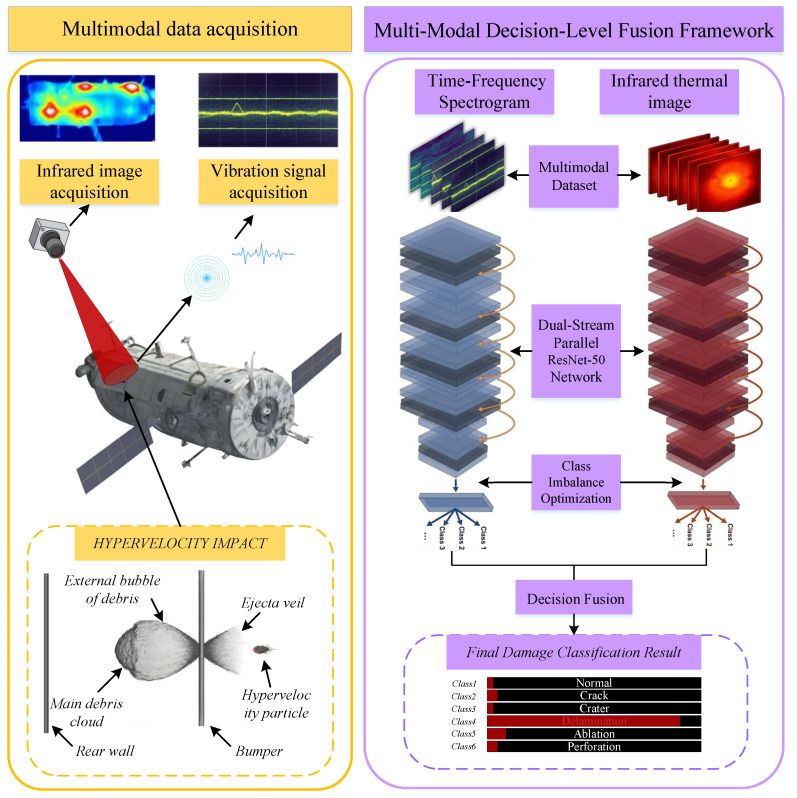
Overall flowchart of the vibration-infrared multimodal fusion classification method.

This study aims to establish a robust multimodal fusion framework for high-precision HVI damage classification. Key objectives include: (1) overcoming unimodal physical limitations, such as vibration insensitivity and thermal blurring; (2) mitigating feature degradation caused by noise and variable imaging distances; and (3) resolving inter-modal conflicts via uncertainty quantification for reliable diagnosis. By integrating physics-informed signal enhancement with evidence-theoretic fusion, this work provides an accurate solution for on-orbit structural health monitoring. As depicted in Figure 1, the proposed framework targets the challenge of high-precision HVI damage classification. Key contributions include:

1. A vibration-infrared multimodal fusion framework that systematically integrates vibration time–frequency analysis with infrared distance-aware enhancement, providing a robust solution for feature extraction in variable environments.
2. A dual-stream deep residual network architecture developed to extract and fuse modality-specific features. It combines spectral subtraction and STFT for vibration signals and utilizes hierarchical enhancement to address infrared feature shifts caused by imaging distance variations.
3. A decision-level fusion method based on D-S evidence theory. By quantifying epistemic uncertainty, this approach suppresses inter-modal conflicts and maximizes complementary advantages, ensuring reliable classification for spacecraft maintenance.

## 2. Problem Statement

**Figure 2:**
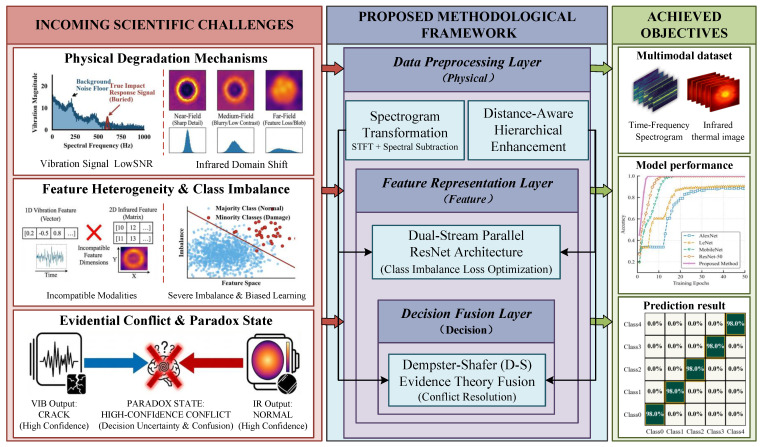
Multi-level challenge-fusion framework for HVI damage classification.

Spacecraft inevitably encounter hypervelocity impacts (HVIs) from micrometeoroids and orbital debris (MMOD). To ensure survivability, mission requirements extend beyond detection to the precise classification of damage types (e.g., cracks, delamination, or perforation) for targeted maintenance. However, transitioning to high-precision classification faces fundamental challenges of intrinsic ambiguity and feature aliasing. As illustrated in Figure 2, these challenges propagate from the physical perception layer to the decision layer.

**First, signal degradation in harsh environments limits sensing reliability.** Vibration responses, critical for identifying micro-damage, are often masked by broadband mechanical noise, resulting in a low Signal-to-Noise Ratio (SNR) that renders time-domain analysis ineffective. Similarly, infrared feature representation is highly sensitive to imaging distance. Variations in detection distance cause severe “domain shift” in thermal feature distributions, hindering model generalization under variable operating conditions.

**Second, data heterogeneity and sample scarcity create a “semantic gap” in feature learning.** Synergizing 1D temporal vibration signals (global stiffness) with 2D thermal images (local flow blockage) requires aligning incompatible geometric manifolds. Furthermore, real-world data exhibits an extreme long-tail distribution: scarce high-risk samples (e.g., perforations) are overwhelmed by abundant minor damage samples. This imbalance biases decision boundaries toward the majority class, leading to critical missed detections.

**Finally, sensor conflicts compromise deterministic decision-making.** In complex scenarios, a “Paradox State” arises when sensors yield contradictory diagnoses (e.g., internal delamination triggering vibration anomalies while remaining thermally invisible). In such cases, traditional hard-voting mechanisms fail. Therefore, establishing a mathematical framework to quantify “epistemic uncertainty” is imperative to manage conflicts and preserve high-confidence evidence.

To systematically address these challenges—signal degradation (Physical Layer), feature alignment gaps (Feature Layer), and evidential conflicts (Decision Layer)—this paper proposes a multimodal fusion framework (Figure 2, right panel) that achieves robust classification through multi-level processing mechanisms. To satisfy on-orbit real-time constraints, the framework prioritizes computational efficiency through a lightweight design. By adopting decision-level fusion over high-dimensional feature concatenation, computational overhead is minimized. The decoupled dual-stream topology supports hardware parallelism, ensuring system latency is determined by the longest single branch rather than cumulative processing time. Additionally, input dimension optimization reduces floating-point operations (FLOPs), guaranteeing rapid response capabilities for sudden impacts.

## 3. Methodology Description

### 3.1. Vibration Signal Enhancement and Time–Frequency Representation

**Figure 3:**
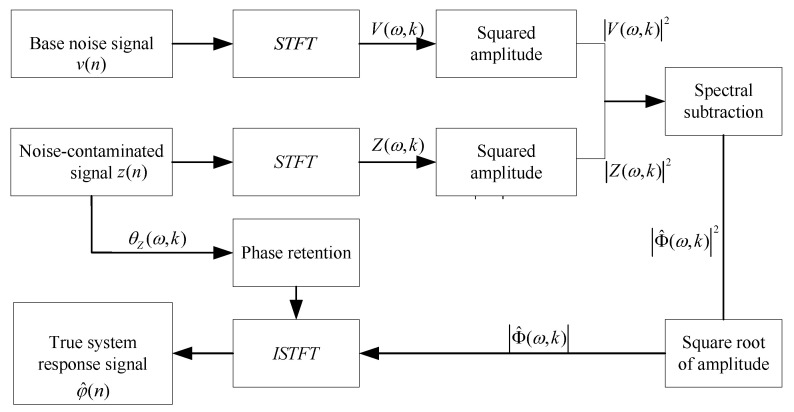
Schematic diagram of the principle of spectral subtraction.

Under ultrasonic broadband excitation, vibrational responses are critical for damage identification but are often compromised by low Signal-to-Noise Ratios (SNR) due to environmental interference. To recover transient damage features, a spectral subtraction algorithm is employed. Crucially, this method suppresses additive noise while preserving original phase information, ensuring the temporal integrity required for accurate time–frequency analysis. The implementation principle is illustrated in Figure 3.

The observed signal z(n) is modeled as a superposition of the true system response φ(n) and additive noise v(n):

(1) z(n)=φ(n)+v(n),1≤n≤N

where *N* is the signal length. Applying the Short-Time Fourier Transform (STFT) transforms the signal into the frequency domain:

(2) Z(ω,k)=Φ(ω,k)+V(ω,k)

Here, *Z*, Φ, and *V* correspond to the frequency representations of the noisy signal, true response, and noise, respectively. These components are decomposed into magnitude and phase:

(3) $$\begin{array}{cc} Z(\omega ,k) & =\left|Z(\omega ,k)\right|\;\cdot \;e^{j\theta_{Z}\left(\omega ,k\right)} \end{array}$$

(4) $$\begin{array}{cc} V(\omega ,k) & =\left|V(\omega ,k)\right|\;\cdot \;e^{j\theta_{V}\left(\omega ,k\right)} \end{array}$$

where |·| denotes magnitude and θ(·) denotes phase.

To recover the damage-related features, Spectral Subtraction is employed. The noise magnitude spectrum $|\hat{V}\left(\omega ,k\right)|$ is estimated from baseline non-excited signals. The denoised amplitude spectrum $|\hat{\Phi }\left(\omega ,k\right)|$ is then obtained by subtracting the noise floor:

(5) $$\left|\hat{\Phi }\left(\omega ,k\right)\right|=\max\left\{\left|Z(\omega ,k)\right|-\left|\hat{V}\left(\omega ,k\right)\right|,0\right\}$$

The time-domain signal $\hat{\varphi }\left(n\right)$ is reconstructed via the Inverse STFT (ISTFT) by combining the denoised magnitude with the original noisy phase $\theta_{Z}\left(\omega ,k\right)$, preserving the temporal characteristics of the impact:

(6) $$\hat{\varphi }\left(n\right)=ISTFT\left[\left|\hat{\Phi }\left(\omega ,k\right)\right|\;\cdot \;e^{j\theta_{Z}\left(\omega ,k\right)}\right]$$

To standardize inputs for deep learning and expand the dataset, an overlapping sliding window strategy is applied to the denoised sequence $\hat{\varphi }\left(n\right)$. Given a window length $W_{L}$ and step size $l_{s}$, the overlap ratio $\rho_{r}$ is defined as:

(7) $$\rho_{r}=\left(1-\frac{l_{s}}{W_{L}}\right)\times 100\%$$

The *l*-th signal segment $\Delta_{l}^{\varphi }$ is extracted as follows:

(8) $$\Delta_{l}^{\varphi }={\left[\hat{\varphi }\left(l\;\cdot \;l_{s}\right),\hat{\varphi }\left(l\;\cdot \;l_{s}+1\right),\ldots ,\hat{\varphi }\left(l\;\cdot \;l_{s}+W_{L}-1\right)\right]}^{T},l=1,2,\ldots ,L_{s}$$

The total number of segments $L_{s}$ is calculated by:

(9) $$L_{s}=\left[\frac{N-W_{L}}{l_{s}}\right]+1$$

In this study, parameters are set to $W_{L}=2048$ and $l_{s}=512$ ($\rho_{r}=75\%$) to capture local features while ensuring continuity. Each segment is assigned a damage label $c_{l}\in \left\{0,1,\ldots ,K_{c}-1\right\}$, constructing the labeled vibration dataset:

(10) $$D_{vib}={\left\{\left(\Delta_{l}^{\varphi },c_{l}\right)\right\}}_{l=1}^{L_{s}}$$

Finally, $D_{\mathrm{vib}}$ is partitioned into training and testing sets (8:2 ratio) using stratified sampling to maintain consistent class distribution and prevent overfitting.

### 3.2. Distance-Aware Data Augmentation for Infrared Images

Infrared imaging quality in spacecraft NDT degrades significantly with varying detection distances. To address this, a distance-aware data augmentation strategy is proposed to physically simulate degradation across near-, mid-, and far-field regions. This approach employs hierarchical enhancement functions $\Gamma_{d}$ to model distinct degradation patterns: $\Gamma_{near}$ enhances high-resolution detail separability; $\Gamma_{mid}$ applies geometric transformations for inter-class discriminability; and $\Gamma_{far}$ compensates for resolution loss and noise.

The enhancement logic is formally defined in Equation (11), where *d* denotes the simulated distance, and $d_{n},d_{f}$ represent the near- and far-field thresholds, respectively.

(11) $$I_{enh}=\left\{\begin{array}{cc} \Gamma_{near}\left(I_{raw}\right) & d<d_{n}\\ \Gamma_{mid}\left(I_{raw}\right) & d_{n}\leq d\leq d_{f}\\ \Gamma_{far}\left(I_{raw}\right) & d>d_{f} \end{array}\right.$$

The complete data generation process is summarized in Algorithm 1. By generating multi-distance samples consistent with physical variations, this method effectively improves model robustness under diverse imaging conditions.
**Algorithm 1** Distance-Aware Infrared Data Augmentation**Require:** Raw infrared images $I_{\mathrm{raw}}$, Augmentation factor $N_{e}$, Thresholds $d_{n},d_{f}$**Ensure:** Augmented dataset $D_{\mathrm{ir}}$, split into $D_{\mathrm{ir\_train}},D_{\mathrm{ir\_test}}$1:Initialize $D_{\mathrm{ir}}\leftarrow \emptyset$2:**for** each image $I\in I_{\mathrm{raw}}$ **do**3:    **for** k=1 to $N_{e}$ **do**4:        Generate simulated distance $d\leftarrow \mathrm{Random}(d_{\min},d_{\max})$5:        **Determine Category** $c_{d}$ based on Equation (11):6:        **if** $d<d_{n}$ **then** $c_{d}\leftarrow \mathrm{Near}$7:        **else if** $d>d_{f}$ **then** $c_{d}\leftarrow \mathrm{Far}$8:        **else** $c_{d}\leftarrow \mathrm{Mid}$9:        **end if**10:        **Apply Transformation:** $I_{\mathrm{enh}}\leftarrow \Gamma_{d}\left(I,c_{d}\right)$11:        $D_{\mathrm{ir}}\leftarrow D_{\mathrm{ir}}\cup \left\{I_{\mathrm{enh}}\right\}$12:    **end for**13:**end for**14:**Partition:** Split $D_{\mathrm{ir}}$ into $D_{\mathrm{ir\_train}}$ (80%) and $D_{\mathrm{ir\_test}}$ (20%) using stratified sampling15:**return** $D_{\mathrm{ir\_train}},D_{\mathrm{ir\_test}}$

### 3.3. Multi-Modal Feature Extraction via a Dual-Stream ResNet

**Figure 4:**
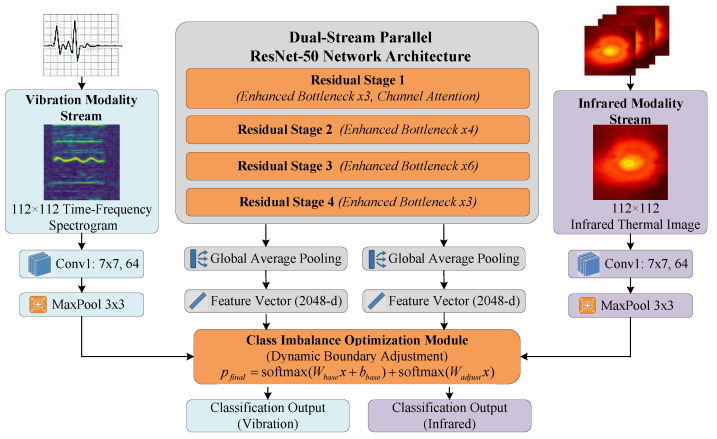
Schematic diagram of the Dual-Stream ResNet network structure.

Building on the preprocessed datasets $D_{vib}$ and $D_{ir}$, we design a dual-stream enhanced ResNet to extract hierarchical features. ResNet-50 is selected as the backbone for its optimal balance between feature abstraction and convergence efficiency under limited data. Its residual learning framework effectively mitigates gradient vanishing by reformulating the mapping H(x) as learning a residual function F(x):

(12) $$H\left(x\right)=F(x,\left\{W_{t}\right\})+x$$

where *x* and $\left\{W_{t}\right\}$ denote the input and trainable parameters, respectively. As shown in Figure 4, the architecture employs two parallel, weight-independent streams to preserve modality-specific representations.

To enhance feature discriminability, a Channel Attention Mechanism is integrated into the ResNet Bottleneck. For an input feature map $\chi \in^{C\times H\times W}$, attention weights *o* are computed via a dual-path pooling strategy comprising Global Average Pooling (GAP) and Global Max Pooling (GMP):

(13) $$\begin{array}{cc} z_{\mathrm{avg}} & =\mathrm{GAP}\left(\chi \right)=\frac{1}{H\times W}\sum_{i=1}^{H}\sum_{j=1}^{W}\chi \left[:,i,j\right] \end{array}$$

(14) $$\begin{array}{cc} z_{\max} & =\mathrm{GMP}\left(\chi \right)=\max_{i,j}\chi \left[:,i,j\right] \end{array}$$

(15) $$\begin{array}{cc} o & =\sigma (W_{2}\delta \left(W_{1}z_{\mathrm{avg}}\right)+W_{2}\delta \left(W_{1}z_{\max}\right)) \end{array}$$

Here, $W_{1}$ and $W_{2}$ are learned weights with a reduction ratio r=8, while δ and σ denote ReLU and Sigmoid functions. The feature map is adaptively refined via element-wise multiplication:

(16) $$\chi_{out}=o⊙\chi$$

Through this element-wise multiplication (⊙), the mechanism adaptively highlights channels critical for damage characterization while suppressing background noise.

To address sample scarcity (particularly in vibration data), a weighted decision layer is introduced to dynamically adjust decision boundaries. The final probability $p_{final}$ fuses a base classifier with an adjustment term:

(17) $$\begin{array}{cc} p_{\mathrm{base}} & =\mathrm{softmax}(W_{\mathrm{base}}x+b_{\mathrm{base}}) \end{array}$$

(18) $$\begin{array}{cc} p_{\mathrm{adjust}} & =\mathrm{softmax}(W_{\mathrm{adjust}}x) \end{array}$$

(19) $$\begin{array}{cc} p_{\mathrm{final}} & =p_{\mathrm{base}}+p_{\mathrm{adjust}} \end{array}$$

where *x* is the high-level feature vector. The adjustment layer $W_{adjust}$ (initialized to zero) specifically learns compensatory weights for minority classes, ensuring unbiased optimization during training.

Crucially, the dual-stream architecture outputs instance-level probability vectors for the subsequent fusion stage. By processing synchronized single-frame inputs, the network yields Softmax outputs ($P_{vib}$ and $P_{ir}$) representing class probabilities. These vectors, distinct from intermediate feature maps or temporal sequences, serve as the precise inputs for the Dempster–Shafer fusion module.

### 3.4. Multi-Modal Decision Fusion Based on D-S Evidence Theory

**Figure 5:**
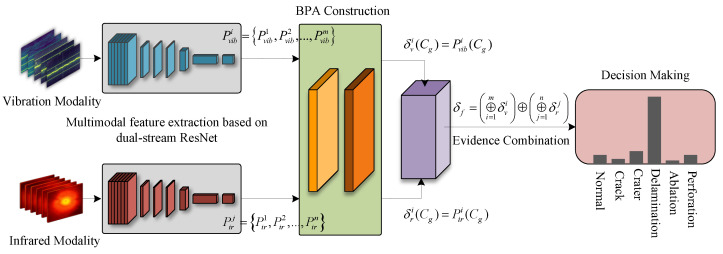
Schematic diagram of the multimodal fusion decision-making method based on Dempster–Shafer evidence theory.

To address the varying reliability of vibration and infrared modalities, we employ Dempster–Shafer (D-S) evidence theory for decision-level fusion (Figure 5). This framework explicitly models epistemic uncertainty, allowing for robust conflict resolution between heterogeneous data sources.

The decision framework is defined over a set of mutually exclusive damage states $\Theta =\{C_{1},C_{2},\ldots ,C_{G}\}$. The core mechanism involves constructing Basic Probability Assignments (BPAs) from network Softmax outputs and fusing them via Dempster’s combination rule. The mathematical definition for combining two independent evidences ($\delta_{1},\delta_{2}$) is given by the orthogonal sum:

(20) $$\delta_{1⊕2}\left(C_{g}\right)=\frac{1}{1-\kappa }\sum_{A\cap B=C_{g}}\delta_{1}\left(A\right)\delta_{2}\left(B\right)$$

where $\kappa =\sum_{A\cap B=\emptyset }\delta_{1}\left(A\right)\delta_{2}\left(B\right)$ represents the conflict coefficient. This rule is associative and can be applied iteratively to fuse multiple evidence sources from both modalities, as outlined in Algorithm 2.
**Algorithm 2** Multi-modal Fusion Decision Method Based on D-S Evidence Theory**Require:**      **Vibration Probabilities:** $P_{vib}^{i}=\left\{P_{vib}^{1},\ldots ,P_{vib}^{m}\right\}$ for i=1…m      **Infrared Probabilities:**    $P_{ir}^{j}=\left\{P_{ir}^{1},\ldots ,P_{ir}^{n}\right\}$ for j=1…n
**Ensure:** Final decision class $c^{*}$1:**Step 1: BPA Construction**2:Map probabilities to BPAs for all sensors/frames:3:$\delta_{v}^{i}\left(\left\{C_{g}\right\}\right)\leftarrow P_{vib}^{i}\left(\left\{C_{g}\right\}\right),\;\forall i\in \left[1,m\right],\forall g\in \left[1,G\right]$4:$\delta_{r}^{j}\left(\left\{C_{g}\right\}\right)\leftarrow P_{ir}^{j}\left(\left\{C_{g}\right\}\right),\;\forall j\in \left[1,n\right],\forall g\in \left[1,G\right]$5:**Step 2: Evidence Combination**6:Fuse all evidence bodies iteratively using Dempster’s rule (Equation (20)):7:$\delta_{f}=\left(\delta_{v}^{1}⊕\delta_{v}^{2}⊕\ldots ⊕\delta_{v}^{m}\right)⊕\left(\delta_{r}^{1}⊕\delta_{r}^{2}⊕\ldots ⊕\delta_{r}^{n}\right)$8:**Step 3: Decision Making**9:Select class with maximum belief:10:$c^{*}\leftarrow \arg\max_{C_{g}\in \Theta }\delta_{f}\left(C_{g}\right)$11:**return** $c^{*}$


## 4. Results and Discussion

### 4.1. Experimental Setup and Dataset Construction

#### 4.1.1. Preparation and Characterization of HVI Damaged Specimens

This study focuses on five typical spacecraft damage types: cracks, craters, delamination, ablation, and perforation, unlike simulation-based studies, this research relies on high-fidelity physical data obtained from a professional two-stage light gas gun ballistic range. To accurately simulate the structural response of modern spacecraft thermal protection systems, the target specimens were manufactured from Carbon Fiber Reinforced Carbon (C/C) composites (100mm×100mm×8mm). Spherical aluminum projectiles with a diameter of 3.0 mm were employed to impact these targets at velocities ranging from 1.5 km/s to 5.0 km/s in a vacuum environment.

High-velocity penetration generated craters and perforations, while shock wave propagation induced internal delamination and cracks. To ensure label reliability, a “physical measurement + expert verification” protocol was implemented. Samples were quantified via optical microscopy (e.g., typical craters: 10.3–10.7 mm diameter) and independently validated by two experts to establish the Ground Truth.

#### 4.1.2. Multimodal Data Acquisition and Preprocessing Strategy

**Figure 6:**
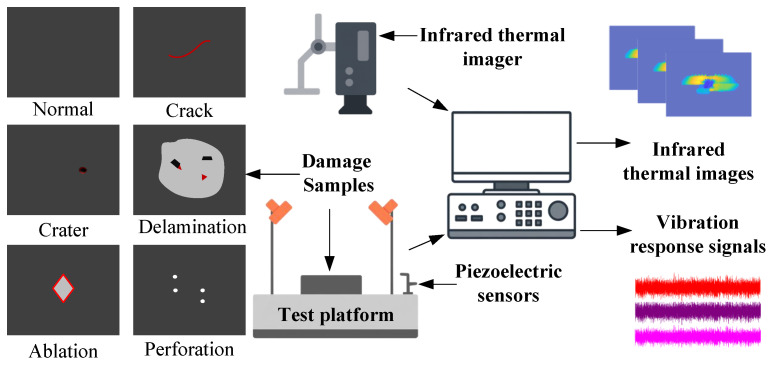
Schematic of the multi-modal data fusion damage classification detection platform.

The system architecture and operational workflow of the experimental platform are illustrated in Figure 6. The infrared thermography system captures the specimen’s two-dimensional temperature distribution, yielding thermal images with a resolution of 640×512 pixels. Concurrently, piezoelectric accelerometers record surface vibration signals at a sampling rate of 250 kHz. To ensure the validity of multimodal fusion, a strict sequential acquisition protocol was implemented. Since HVI damage constitutes a permanent plastic deformation with static stability, strict temporal synchronization is not required. By sequentially capturing thermal and vibration data from the same damaged specimen, physical state consistency is maintained, ensuring that both modalities characterize the identical damage event for decision-level fusion.

In the preprocessing stage, specific strategies were employed to enhance signal quality and standardize inputs. Regarding the sensitivity to non-stationary noise, although standard spectral subtraction assumes stationarity, our method remains robust due to the extremely short duration of HVI response. Within this micro-to-millisecond scale, the statistical properties of background noise (such as thermal noise and mechanical micro-vibrations) can be approximated as quasi-stationary. Consequently, the signal processing is conducted within STFT frames (256 points, approx. 1 ms), where noise properties remain stable. Furthermore, the subsequent ResNet backbone focuses on the topological structure of energy ridges rather than absolute pixel amplitudes, providing feature-level tolerance to residual noise. The signals were converted into time–frequency spectrograms using a Hanning window of length 256, a hop size of 64, and 256 FFT points, followed by a logarithmic amplitude scale and robust Min-Max normalization (based on 5th and 95th percentiles). Subsequently, both inputs were resized to 112×112 pixels. This resolution was selected to balance accuracy and efficiency: (1) It adapts to constrained on-orbit computing resources by reducing FLOPs. (2) It preserves sufficient physical fidelity, specifically the low-frequency energy ridges in vibration spectra and thermal gradients in infrared images. (3) It ensures network compatibility, as the size is divisible by the ResNet-50 downsampling factor, maintaining spatial resolution in the final feature maps.

To validate the proposed method, a multi-modal dataset was constructed covering six states: Class 0 (Normal), Class 1 (Crack), Class 2 (Crater), Class 3 (Delamination), Class 4 (Ablation), and Class 5 (Perforation). As shown in Table 1, the dataset exhibits inherent class imbalance due to the physical rarity of high-velocity impact samples. To establish robustness under this imbalance, we introduced a class-weighted Cross-Entropy loss mechanism to assign higher gradient weights to rare samples. Simultaneously, strictly regularized optimization strategies, including the AdamW optimizer with weight decay and a Cosine Annealing learning rate schedule, were implemented to restrict the model from falling into sharp local minima and prevent overfitting. Crucially, to prevent data leakage, an event-based splitting strategy was implemented instead of random shuffling. Samples were partitioned based on the unique Experiment ID at a ratio of 7:2:1. This ensures that the test set consists of completely unseen physical events, effectively assessing the model’s true generalization capability.

#### 4.1.3. Hyperparameter Settings and Evaluation Metrics

Experiments were conducted on a Windows 10 workstation equipped with an Intel i5-12400 CPU, 16 GB RAM, and an NVIDIA GTX 1650 SUPER GPU. All algorithms were implemented using Python 3.7 and PyTorch 1.7.1. To ensure reproducibility, the specific network training hyperparameters (including the AdamW optimizer and Cosine Annealing strategy) are summarized in the upper section of Table 2.

To comprehensively quantify the model performance, a multi-dimensional evaluation metric system was established, as mathematically defined in the lower section of Table 2. First, to evaluate the efficacy of the proposed vibration spectrogram preprocessing, we introduced a custom feature separability score ($\lambda_{sep}$). This metric quantifies the statistical contrast between the signal energy ridges and the background noise, providing a direct measure of signal-to-noise ratio enhancement. Regarding classification performance, relying solely on Accuracy is insufficient due to the inherent class imbalance in HVI datasets. Therefore, we incorporated Precision, Recall, and F1-Score to analyze class-specific performance. More importantly, to assess global robustness, we employed Macro-F1 and Cohen’s Kappa coefficient. Macro-F1 calculates the unweighted average across all categories, ensuring that minority classes (e.g., Perforation) contribute equally to the final score. Cohen’s Kappa evaluates the agreement between predictions and ground truth after removing the possibility of chance agreement, thereby verifying that the high accuracy is not a result of statistical bias toward majority classes.

### 4.2. Quantitative Validation of Signal Reconstruction and Enhancement Modules

This section quantitatively verifies the independent contributions of the proposed preprocessing modules—Spectral Subtraction (SS) for vibration and Distance-Aware Augmentation (DA) for infrared—ensuring that the high performance of the fusion system stems from high-quality unimodal features rather than the masking effect of the decision mechanism.

#### 4.2.1. Efficacy of Spectral Subtraction Denoising

**Figure 7:**
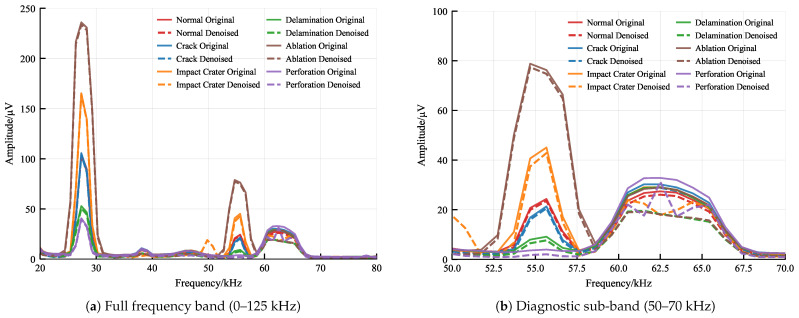
Spectral comparison demonstrating the denoising efficacy. (**a**) The full-band spectrum shows global noise suppression. (**b**) The zoomed diagnostic band (50–70 kHz) reveals that spectral subtraction effectively removes high-frequency background noise, highlighting the damage-related resonant peaks.

To visualize the signal reconstruction capability, Figure 7 presents the frequency-domain analysis. Original signals (blue) are heavily contaminated by environmental noise in the 60–70 kHz band, masking the true damage signatures. The proposed spectral subtraction (orange) effectively suppresses this noise floor while preserving the discriminative spectral peaks at 30 kHz and 55 kHz, significantly improving the Signal-to-Noise Ratio (SNR).

**Figure 8:**
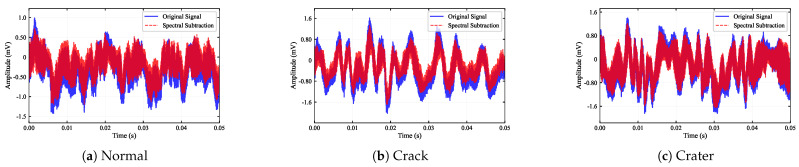

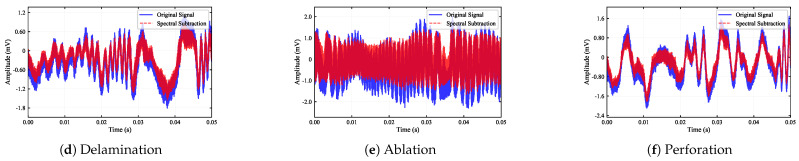
Time-domain signal reconstruction across six material states. The red curves indicate the denoised signals, which retain the dominant high-amplitude shock components while filtering out low-amplitude baseline fluctuations compared to the original signals (blue).

The time-domain characteristics are detailed in Figure 8. Comparing the raw and denoised waveforms, it is evident that the stochastic background micro-vibrations are attenuated. For complex damage types like Crater and Ablation, the main impact pulse peaks (approx. 1.6 mV) become distinguishable from the clutter, verifying that the subtraction process does not erode the transient shock features.

**Figure 9:**
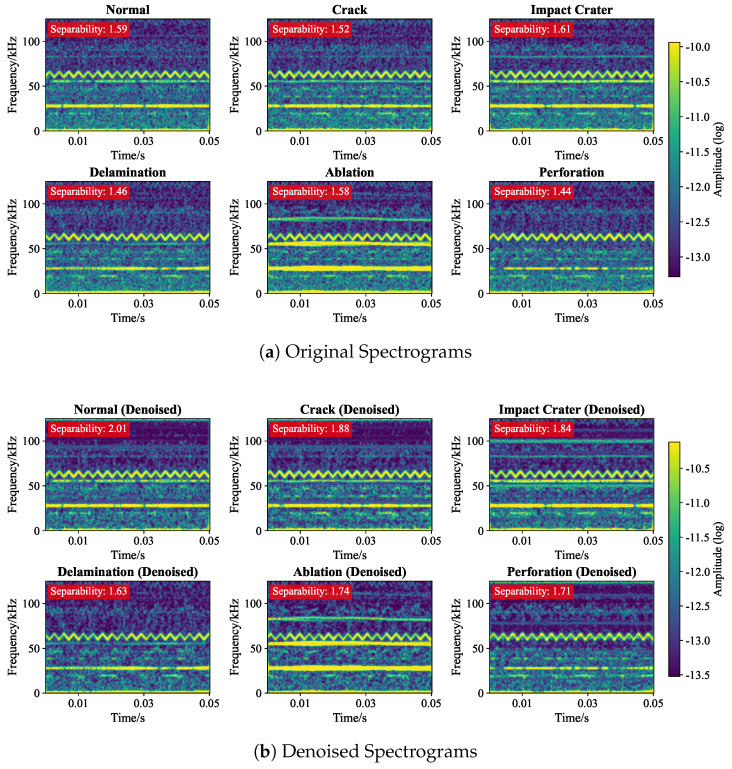
Comparison of Time–Frequency Spectrograms. (**a**) Original inputs suffer from smearing noise. (**b**) Denoised outputs exhibit clear energy ridges. The improved $\lambda_{sep}$ values demonstrate enhanced feature separability.

Furthermore, Figure 9 contrasts the Time–Frequency (TF) spectrograms. The separability scores ($\lambda_{sep}$) marked in red quantify the contrast improvement. For the Normal state, $\lambda_{sep}$ increases from 1.59 to 2.44, indicating a cleaner background. Crucially, for Perforation, the denoising reveals hidden high-frequency transient bursts that were previously submerged in noise, validating the necessity of SS for feature extraction.

#### 4.2.2. Efficacy of Distance-Aware Infrared Augmentation

**Figure 10:**
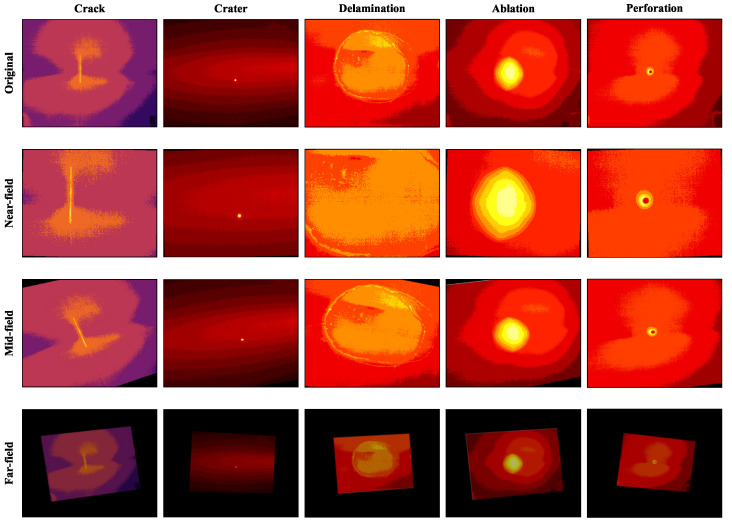
Visual matrix of the distance-aware data augmentation strategy. Rows correspond to Near, Mid, and Far fields, physically modeling the radiometric and geometric distortions encountered in orbit.

Figure 10 visually demonstrates the proposed Distance-Aware Augmentation (DA) strategy. To ensure reproducibility and address the domain shift caused by varying imaging distances, we established specific mathematical definitions for the transformation zones based on the scaling factor S∝1/d (where S=1.0 is the baseline). Specifically, the Near-field zone ($\Gamma_{near}$), defined by the threshold S>1.2, simulates close-range inspection through Scale Up and Crop operations to emphasize the high-frequency textures of micro-cracks. The Mid-field zone ($\Gamma_{mid}$), covering the range 0.8≤S≤1.2, represents standard monitoring conditions, utilizing Random Rotation ($\pm 15^{\circ }$) and perspective distortion to model probe angle deviations. Finally, the Far-field zone ($\Gamma_{far}$), where S<0.8, simulates remote detection by applying Scale Down and Pad operations, coupled with Gaussian Blur (radius r=3) and Gaussian Noise (σ=0.05) to replicate atmospheric attenuation and low-SNR degradation.

#### 4.2.3. Ablation Study Results

To rigorously quantify the contribution of these modules, independent ablation studies were conducted on single-modality networks, as detailed in Table 3. In the vibration modality, the baseline model using raw STFT signals achieved an accuracy of only 51.67% due to severe noise interference. However, the application of Spectral Subtraction (SS) yielded a substantial performance boost, increasing accuracy by 44.16% to reach 95.83%. This confirms that denoising is a prerequisite for effective vibration feature extraction. Similarly, for the infrared modality, the proposed Distance-Aware (DA) strategy was compared against standard geometric augmentation. The DA method improved accuracy from 87.78% to 98.33% (a gain of +10.55%). This significant improvement verifies that physically modeling domain shifts, such as blur and scale variations, enables the network to learn robust features invariant to imaging distances.

### 4.3. Performance Comparison of Classification Models and Robustness Verification

This section evaluates the classification performance, establishing unimodal baselines before demonstrating the advantages of multimodal fusion. The analysis proceeds from training convergence verification to a rigorous quantitative comparison against state-of-the-art benchmarks.

**Figure 11:**
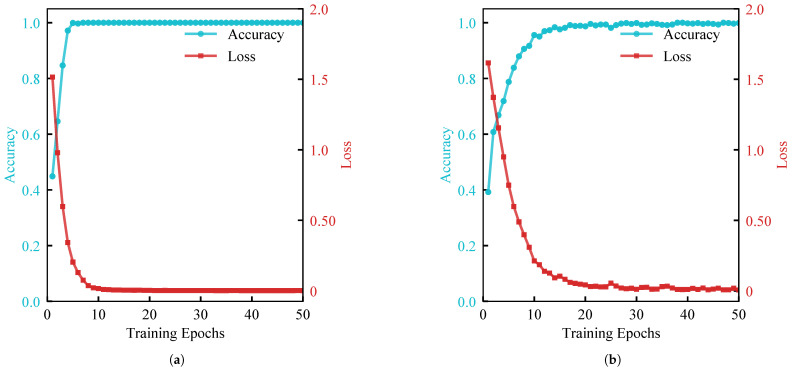
Comparative analysis of training performance between vibration and infrared modalities for spacecraft damage classification. (**a**) Vibration modality. (**b**) Infrared modality.

The training progress, illustrated in Figure 11, indicates that both vibration and infrared branches effectively learn discriminative features. The loss function decreases rapidly approaching zero, and accuracy stabilizes near the optimum after approximately 10 epochs. These synchronized convergence trends validate the independent efficacy of both vibration and infrared inputs for damage recognition tasks.

#### 4.3.1. Comparative Assessment of Unimodal Baselines

**Figure 12:**
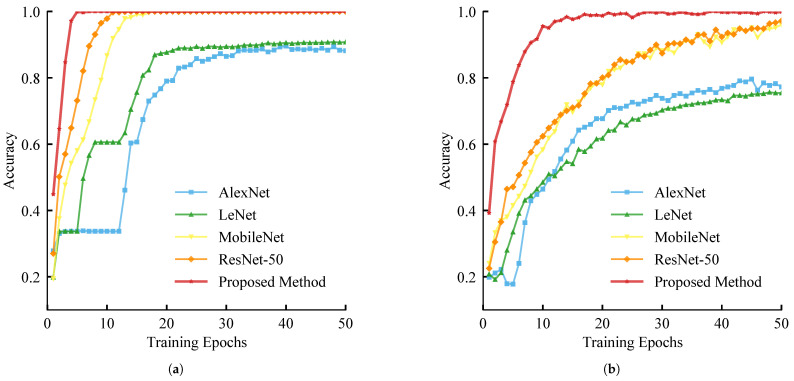
Comparative analysis of training performance for different classification models across vibration and infrared modalities. (**a**) Vibration modality. (**b**) Infrared modality.

To assess architectural superiority, we evaluated the proposed method against four benchmarks, with quantitative results detailed in Table 4. As indicated by the training dynamics (Figure 12), the proposed dual-stream backbone exhibits the fastest convergence and highest stability, effectively overcoming the instability observed in MobileNetV2 (vibration stream) and the fluctuations in lightweight models (infrared stream).

Quantitatively, the proposed method establishes state-of-the-art baselines in both modalities. In the vibration domain, it attains 95.83% accuracy and 94.61% Kappa, significantly outperforming MobileNetV2 and classical CNNs by maintaining a balanced Precision-Recall profile (∼95%). In the infrared domain, compared with other baseline methods, the proposed method maintains excellent classification results (98.33% accuracy, 98.34% F1-Score). These results confirm the backbone’s robustness in extracting discriminative features from both high-frequency impact signals and thermal images.

#### 4.3.2. Architectural Optimization: Attention Mechanism Analysis

To validate the rationale behind inserting the Channel Attention Mechanism (CAM) at the shallow bottleneck (Stage 1), we conducted a comparative ablation study against variants with no attention (Baseline), high-level attention (Stage 4), and full-stage attention. As shown in Table 5, the proposed Stage-1 strategy achieves optimal results in both modalities. Notably, placing attention only at high levels (Stage 4) resulted in negligible improvement or even degradation. From a physical perspective, this confirms that HVI damage is characterized by low-level texture features (e.g., micro-cracks) rather than high-level semantics. Enhancing these bottom-level features is crucial for identifying micro-damage signals in imbalanced datasets, further supporting the architectural rationality against overfitting concerns.

To further elucidate the impact on minority classes, Table 6 details the per-class metrics for the vibration modality. The High-level model fails to capture weak features, resulting in a Recall of only 0.5469 for the Crack class. In contrast, the Proposed method significantly improves the Crack Recall to 0.7656 and achieves perfect recognition (Recall 1.0) for Delamination and Ablation. This confirms that enhancing low-level feature extraction is essential for identifying subtle damage signals in imbalanced datasets.

#### 4.3.3. Generalization Assessment via Distance-Domain Cross-Validation

To rigorously assess the model’s robustness against domain shifts, we conducted a targeted generalization test. While vibration signals exhibit high consistency across specimens due to contact measurement, the infrared modality involves non-contact imaging where distance variations introduce severe geometric scaling, serving as a stringent proxy for out-of-distribution (OOD) generalization. We employed a “Leave-One-Distance-Out” protocol, partitioning the dataset into distinct Near, Mid, and Far-field domains.

The cross-validation results in Table 7 confirm the model’s robustness. In Scenarios 2 and 3, where the training set included distance anchors (e.g., Near & Far), the model successfully generalized to the unseen Mid-field domain, achieving 76.11% accuracy. This capability to perform well on physically distinct domains with different noise distributions proves that the model learns intrinsic damage representations invariant to scale and blur. Consequently, the high training accuracy represents excellent fitting to these physical laws rather than memorization of background noise.

### 4.4. Synergistic Efficacy of Multimodal Decision-Level Fusion

This section quantitatively validates the synergistic effect of the D-S fusion mechanism. By integrating complementary evidence from vibration and infrared sensors, the fusion framework aims to overcome the physical limitations inherent in single-modality perception.

#### 4.4.1. Category-Wise Performance Improvement

**Figure 13:**
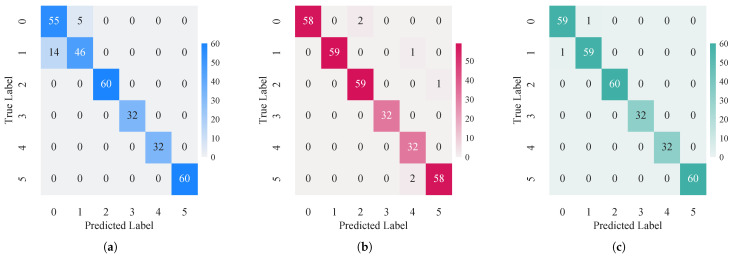
Comparative analysis of confusion matrices. (**a**) Vibration modality. (**b**) Infrared modality. (**c**) Fusion decision result.

**Figure 14:**
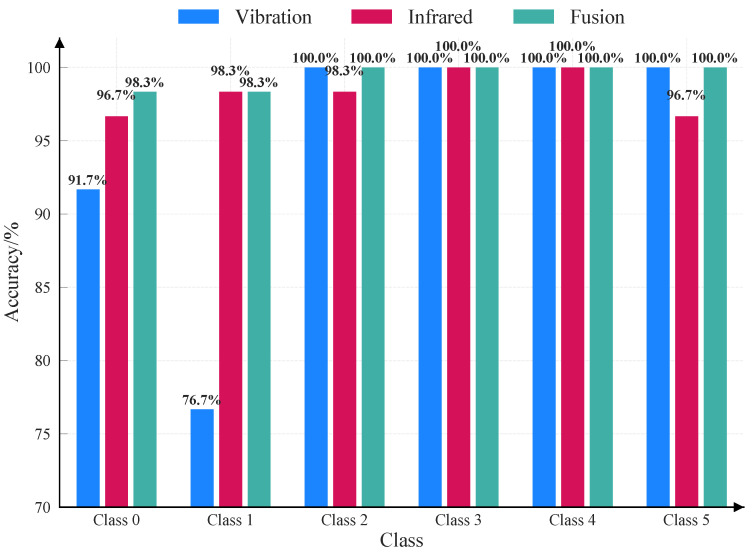
Comparison results of accuracy rates for each damage category.

The visualization results in Figure 13 and Figure 14 illustrate the class-specific performance gains. The confusion matrices (Figure 13) reveal that unimodal approaches suffer from distinct misclassifications due to spectral or physical limitations. specifically, the vibration modality (Figure 13a) exhibits reduced sensitivity to “Crack” categorie, achieving only 76.7% accuracy for both. This shortfall is likely due to the subtle structural changes induced by “Crack”, which generate weaker global vibration responses compared to more severe damages like perforation.

Conversely, the infrared modality (Figure 13b) demonstrates strong texture recognition capabilities but exhibits slight instability in identifying “Perforation” (96.7%). The proposed D-S fusion strategy (Figure 13c) effectively synthesizes these complementary inputs. It not only matches the superior infrared performance for “Crack” and “Ablation” (recovering to 98.3% and 100.0%) but also corrects the infrared instability in “Perforation” cases. Consequently, the fusion method maintains a robust accuracy range of 96.7–100.0% across all categories, this confirms that the evidence-theoretic fusion successfully mitigates unimodal blind spots, ensuring high-reliability diagnosis.

#### 4.4.2. Comparative Analysis of Fusion Strategies

To demonstrate the superiority of the evidence-theoretic approach under bandwidth-constrained on-orbit conditions, we benchmarked the Proposed D-S method against three standard decision-level fusion strategies: Average Fusion, Max-Confidence Fusion, and Weighted Fusion. The comparative results are presented in Table 8.

In the Normal scenario (full test set), all fusion strategies achieve high accuracy (>99%), exhibiting a ceiling effect due to the strong baseline performance. However, distinct performance gaps emerge in the “High Conflict” scenario (where unimodal predictions disagree). While Average Fusion maintains high accuracy through smoothing, its Precision drops to 0.8750. In contrast, the Proposed D-S method achieves the highest Precision (0.9476) and Macro-F1 (0.9476). This indicates that the D-S evidence theory offers a more rigorous mechanism for handling conflicting sensor inputs, reducing the risk of false positives which is critical for autonomous decision-making in space.

#### 4.4.3. Robustness and Stability Verification

To assess the statistical reliability of the proposed framework, we conducted repeated experiments using five distinct random seeds (42, 123, 888, 2026, 3407) to account for variations in data partitioning and weight initialization. Table 9 reports the mean and standard deviation for key performance metrics.

The statistical results confirm that the fusion framework significantly enhances both accuracy and stability. The fusion method achieves a mean accuracy of 99.01% with a standard deviation of only 0.74%, which is substantially lower than the volatility observed in the single vibration modality (±2.34%). Furthermore, the consistent high performance across randomized data partitions rules out specific bias to a fixed training set, confirming that the model has learned generalized damage features rather than overfitting to specific samples.

## 5. Discussion

### 5.1. Physical Interpretation of Misclassification Mechanisms

The misclassification patterns observed in the confusion matrices stem from the inherent physical limitations of single sensing modalities and the strong physical co-occurrence of HVI damage. In the vibration modality, the misidentification of micro-cracks as normal states arises from the global nature of modal responses; micro-scale defects often fail to induce statistically significant global stiffness changes or frequency shifts, creating a detection blind spot. Similarly, the infrared modality is constrained by thermal diffusion effects, where ablation and delamination generate indistinguishable surface temperature gradients due to similar thermal resistance boundaries under transient heating.

These physical ambiguities confirm that relying on a single sensor inevitably leads to decision uncertainty. By leveraging Dempster–Shafer (D-S) evidence theory, the proposed framework effectively resolves these conflicts. Unlike traditional weighted averaging, which risks propagating errors in high-conflict scenarios, the D-S rule utilizes the “uncertainty” mass to discount conflicting evidence, thereby achieving superior reliability in distinguishing physically coupled damage types.

### 5.2. Framework Scalability and Material Universality

The validity of this study is underpinned by high-fidelity physical experiments rather than numerical simulations. From a mechanistic perspective, the proposed detection framework exhibits theoretical scalability across different material systems. In vibration analysis, damage-induced local mass loss and stiffness degradation inevitably lead to eigenfrequency shifts, a phenomenon governing both metallic and composite structures. Similarly, infrared detection relies on thermal wave scattering at defect interfaces, a fundamental heat transfer behavior applicable to various materials. Therefore, while signal amplitudes may vary, the underlying feature extraction and D-S fusion logic remain transferable. Future applications on other spacecraft materials would primarily require parameter fine-tuning rather than architectural reconstruction.

### 5.3. Limitations and Future Work

Despite the demonstrated efficacy of the proposed framework in identifying damage categories, this study focuses primarily on discrete classification and has not yet addressed the quantitative assessment of damage severity (e.g., hole size or crack depth). The current model provides a qualitative diagnosis suitable for triggering alarms but lacks the capability for precise lifespan prediction. Future work will extend this architecture from classification to regression tasks, aiming to establish a continuous mapping between multimodal features and quantitative damage metrics, thereby providing a more comprehensive solution for spacecraft structural health monitoring.

## 6. Conclusions

This study proposes a physics-informed multimodal fusion framework for reliable spacecraft HVI damage monitoring. By integrating vibration spectral subtraction and distance-aware infrared enhancement within a dual-stream ResNet architecture, the method overcomes unimodal physical limitations such as stiffness insensitivity and thermal ambiguity. A core innovation is the Dempster–Shafer (D-S) evidence-theoretic mechanism, which resolves inter-modal conflicts at the decision level.

Experiments on multi-class HVI specimens demonstrate that the fusion strategy achieves a mean accuracy of 99.01%, significantly outperforming unimodal baselines (92.96% and 97.11%). The D-S mechanism effectively corrects conflicting evidence, achieving perfect precision in four categories. Framework stability is confirmed by a low standard deviation of 0.74% in 5-fold random seed tests, while “Leave-One-Distance-Out” cross-validation proves robust generalization across imaging distances. Furthermore, ablation studies verify that the shallow-layer Channel Attention Mechanism is critical for capturing micro-scale damage features.

In summary, this work establishes a high-precision baseline for on-orbit diagnosis. Future research will extend the framework from discrete classification to continuous regression for quantitative damage assessment and further optimize the model for satellite edge computing deployment.

## Figures and Tables

**Table 1:** Detailed Class Distribution in Multimodal Dataset (Partitioned by Event-based Splitting).

Modality	Subset	Damage Class (Samples)					
		**0**	**1**	**2**	**3**	**4**	**5**
Vibration	Training	256	256	640	128	128	512
	Test	64	64	160	32	32	128
Infrared	Training	220	220	220	220	220	220
	Test	60	60	60	60	60	60
Fusion	Test	60	60	60	32	32	60

**Table 2:** Summary of Experimental Hyperparameters and Evaluation Metric Definitions.

Category	Item	Value/Formula
**Hyperparameters**	Input Size	112×112
	Optimizer	AdamW (Weight decay: $1\times 10^{-3}$)
	Learning Rate	$1\times 10^{-5}$ (Strategy: Cosine Annealing)
	Batch Size	32
	Total Epochs	50
	Random Seed	42, 123, 888, 2026, 3407
**Evaluation Metrics**	Feature Separability	$\lambda_{sep}=\frac{\left|\mu_{f}-\mu_{b}\right|}{\sigma_{f}+\varepsilon }$
		($\mu_{f,b}$: mean pixel intensities, $\sigma_{f}$: feature compactness)
	Accuracy	$\mathrm{Acc}=\frac{TP+TN}{TP+TN+FP+FN}$
	Precision	$\mathrm{Precision}=\frac{TP}{TP+FP}$
	Recall	$\mathrm{Recall}=\frac{TP}{TP+FN}$
	F1-Score	$F1=2\times \frac{P\times R}{P+R}$
		(*P*: Precision, *R*: Recall)
	Macro-F1	$\mathrm{Macro}\text{-}F1=\frac{1}{N}\sum_{i=1}^{N}{F1}_{i}$
		(*N*: number of classes)
	Cohen’s Kappa	$\kappa =\frac{p_{o}-p_{e}}{1-p_{e}}$
		($p_{o}$: observed acc., $p_{e}$: expected chance acc.)

**Table 3:** Independent ablation studies demonstrating the impact of preprocessing modules on Vibration and Infrared modalities.

Configuration	Preprocessing Method	Accuracy	Precision	Macro-F1
***Experiment I: Vibration Modality (Effect of Denoising)***				
Baseline	Raw Signal (STFT only)	51.67%	53.35%	41.41%
Proposed	With Spectral Subtraction (SS)	95.83%	96.06%	94.76%
*Gain*		*+44.16%*	*+42.71%*	*+53.35%*
***Experiment II: Infrared Modality (Effect of Augmentation)***				
Baseline	Standard Geom. Augmentation	87.78%	87.80%	87.65%
Proposed	Distance-Aware Augmentation (DA)	98.33%	98.38%	98.34%
*Gain*		*+10.55%*	*+10.58%*	*+10.69%*

**Table 4:** Performance comparison of different classification models on Vibration and Infrared modalities (Metrics: %).

Model	Vibration Modality					Infrared Modality				
	**Acc**	**Prec**	**Rec**	**F1**	**Kappa**	**Acc**	**Prec**	**Rec**	**F1**	**Kappa**
AlexNet	91.25	89.23	87.89	88.51	88.68	65.56	68.16	65.56	65.33	58.67
LeNet	91.67	92.86	82.55	82.20	89.12	61.11	74.92	61.11	60.29	53.33
MobileNetV2	66.46	58.31	50.81	49.92	56.60	74.44	78.50	74.44	74.46	69.33
ResNet-50	86.04	83.59	73.65	72.22	81.88	78.06	83.43	78.06	78.78	73.67
Proposed	95.83	95.08	94.79	94.76	94.61	98.33	98.38	98.33	98.34	98.00

**Table 5:** Ablation study on Channel Attention Mechanism position. Comparison of global metrics across Vibration and Infrared modalities (Metrics: %).

Model Variant	Attn. Pos.	Vibration Stream			Infrared Stream		
		**Acc**	**Macro-F1**	**Kappa**	**Acc**	**Macro-F1**	**Kappa**
Baseline	None	90.83	87.56	88.11	95.00	95.02	94.00
High-level	Stage 4	91.25	88.01	88.67	92.50	92.61	91.00
Full Version	All Stages	95.83	94.77	94.61	96.67	96.72	96.00
Proposed	Stage 1	95.83	94.76	94.61	98.33	98.34	98.00

**Table 6:** Detailed per-class performance comparison (Precision, Recall, F1-Score) for the Vibration modality.

Damage Class	Baseline (No Attn)			High-Level (Stage 4)			Proposed (Stage 1)		
	**Prec**	**Rec**	**F1**	**Prec**	**Rec**	**F1**	**Prec**	**Rec**	**F1**
Normal (Class 0)	0.6667	0.8438	0.7448	0.6477	0.8906	0.7500	0.7973	0.9219	0.8551
Crack (Class 1)	0.8444	0.5938	0.6972	0.8140	0.5469	0.6542	0.9074	0.7656	0.8305
Crater (Class 2)	0.9755	0.9938	0.9845	1.0000	1.0000	1.0000	1.0000	1.0000	1.0000
Delamination (Class 3)	0.9355	0.9062	0.9206	0.9643	0.8438	0.9000	1.0000	1.0000	1.0000
Ablation (Class 4)	0.9655	0.8750	0.9180	1.0000	0.9688	0.9841	1.0000	1.0000	1.0000
Perforation (Class 5)	0.9771	1.0000	0.9884	0.9846	1.0000	0.9922	1.0000	1.0000	1.0000
Weighted Avg.	0.9139	0.9083	0.9066	0.9217	0.9125	0.9108	0.9606	0.9583	0.9581

**Table 7:** Performance evaluation under strict distance-based generalization scenarios (Metrics: %).

Scenario	Training Domain	Testing Domain	Acc	Prec	Rec	F1	Kappa
Scenario 1	Near & Mid-field	Far-field	36.67	49.04	36.67	32.16	24.00
Scenario 2	Near & Far-field	Mid-field	76.11	79.86	76.11	75.30	71.33
Scenario 3	Mid & Far-field	Near-field	78.89	84.23	78.89	76.74	74.67

**Table 8:** Quantitative comparison of fusion strategies under Normal and High-Conflict scenarios (Metrics: % for Acc, decimal for others).

Scenario	Metric	Average	Max	Weighted	Proposed D-S
Normal Data	Acc	99.67	99.34	99.34	99.34
	Macro-F1	0.9972	0.9944	0.9944	0.9944
	Prec	0.9973	0.9946	0.9945	0.9944
High Conflict	Acc	96.00	92.00	92.00	92.00
	Macro-F1	0.8974	0.8333	0.6808	0.9476
	Prec	0.8750	0.8333	0.6667	0.9476

**Table 9:** Quantitative robustness evaluation across 5 random seed experiments. Results are reported as Mean ± Standard Deviation.

Metric	Vibration	Infrared	Proposed Fusion
Acc	92.96±2.34	97.11±0.86	99.01±0.74
Macro-F1	0.9398±0.020	0.9752±0.006	0.9917±0.006
Prec	0.9445±0.016	0.9756±0.005	0.9919±0.006

## Data Availability

The data presented in this study are available upon request from the corresponding author. The data are not publicly available due to privacy.
